# Editorial: Utilization of healthcare services for children in low and middle-income countries: Its determinants and child health outcomes

**DOI:** 10.3389/fped.2022.1014775

**Published:** 2022-10-25

**Authors:** Bhaskar Thakur, Mona Pathak

**Affiliations:** ^1^ Department of Family and Community Medicine, UT Southwestern Medical Center, Dallas, TX, United States; ^2^ Department of Population and Data Sciences, UT Southwestern Medical Center, Dallas, TX, United States

**Keywords:** healthcare services utilization, child-health outcome, immunization, social determinants, health-inequality, low- and middle-Income countries (LMICs)

**Editorial on the Research Topic** Utilization of healthcare services for children in low and middle-income countries: Its determinants and child health outcomes By Thakur B and Pathak M. (2022) Front. Pediatr. 10: 1014775. doi: 10.3389/fped.2022.1014775

Over the past two decades, the children's health systems worldwide have started paying increasing attention to the healthcare infrastructure, especially in low- and middle-income countries, by debating and focusing more on disease prevention rather than curative care approaches. Even though a declining global trend in child morbidity and mortality has been observed, a recent under-5 mortality rate estimate of 64.6 per 1,000 live births from all 137 low- and middle-income countries (excluding China) is still far from sustainable development goals (SDGs) (1). Proper utilization of services in functional healthcare systems that can prevent such events depends on various critical issues, including quality and accessibility of the current healthcare system, timely intervention, geography, cost, insurance, policy, and other social determinants. The unavailability of complete healthcare infrastructure and underutilization of available functional healthcare systems is the foremost cause of poor child health quality and premature death in low and middle-income countries. Further, healthcare expenditure is another potential barrier to health services utilization in low- and middle-income countries.
